# New sights of immunometabolism and agent progress in colitis associated colorectal cancer

**DOI:** 10.3389/fphar.2023.1303913

**Published:** 2024-01-11

**Authors:** Jingyue Zhang, Chaoyue Chen, Wei Yan, Yu Fu

**Affiliations:** ^1^ Department of Gastroenterology, Union Hospital, Tongji Medical College, Huazhong University of Science and Technology, Wuhan, China; ^2^ Department of Gastroenterology, Tongji Hospital, Tongji Medical College, Huazhong University of Science and Technology, Wuhan, China

**Keywords:** colitis associated colorectal cancer, immunometabolism, metabolic reprogramming, metabolites, inflammation, immune adaption, tumor microenvironment

## Abstract

Colitis associated colorectal cancer is a disease with a high incidence and complex course that develops from chronic inflammation and deteriorates after various immune responses and inflammation-induced attacks. Colitis associated colorectal cancer has the characteristics of both immune diseases and cancer, and the similarity of treatment models contributes to the similar treatment dilemma. Immunometabolism contributes to the basis of life and is the core of many immune diseases. Manipulating metabolic signal transduction can be an effective way to control the immune process, which is expected to become a new target for colitis associated colorectal cancer therapy. Immune cells participate in the whole process of colitis associated colorectal cancer development by transforming their functional condition via changing their metabolic ways, such as glucose, lipid, and amino acid metabolism. The same immune and metabolic processes may play different roles in inflammation, dysplasia, and carcinoma, so anti-inflammation agents, immunomodulators, and agents targeting special metabolism should be used in combination to prevent and inhibit the development of colitis associated colorectal cancer.

## 1 Introduction

Colorectal cancer (CRC) is one of the most common types of fatal malignant tumors around the world (Cassidy and Syed, 2017), mainly including hereditary, sporadic and colitis-related carcinogenesis. It ranks as the second most prevalent cancer detected in women and the third most prevalent in males (Lucas et al., 2017; Dekker et al., 2019; Fernández-Montes et al., 2020). Among them, the colitis associated colorectal cancer (CAC) accouts for approximately 5% of CRC (Zhou and Fang, 2019). CAC arises from a persistent state of inflammation, particularly in the context of inflammatory bowel disease (IBD), which has been consistently increasing in both incidence and prevalence over time (Mak et al., 2020; Birch et al., 2022).

Neoplastic lesions, which have a high chance of developing into cancer, originate as a result of ongoing stimulation by immune cells and the cytokines they release. These lesions can be negative for dysplasia, indeterminate, or classified as low-grade dysplasia (LGD) or high-grade dysplasia (HGD). The process of carcinogenesis in these lesions may be inconsistent or incomplete (Huang and Merchea, 2017). Immunometabolism, a burgeoning discipline, investigates the interplay between immunity and metabolism in various disorders, including metabolic syndrome, neurodegenerative diseases, autoimmune diseases, and cancer.

Immunometabolism can be utilized to illustrate the development of CAC and provide novel perspectives on CAC therapy. During the phase of chronic inflammation marked by a lack of oxygen, caused by different forms of oxidative stress, immune cells generate significant quantities of pro-inflammatory cytokines and metabolites. Consequently, these substances alter their metabolic pathways, intensify the level of inflammation, and trigger mutations in cancer-causing genes. Subsequently, dysplasia arises in the intestinal epithelial cells, triggering cellular growth and various forms of cell death, so exacerbating the disease. Once the immunosuppressive tumor microenvironment (TME) is established, tumor cells can evade immune detection and alter their energy metabolism to accommodate the scarcity of glucose and the build-up of metabolic waste (Dingding et al., 2017; Certo et al., 2021).

Immune modulation serves distinct functions at various stages of CAC, underscoring the significance of comprehending CAC progression accurately and selecting suitable pharmaceutical interventions (Zhang et al., 2019). During the chronic inflammation stage, the administration of anti-inflammatory and anti-immune medications may help prevent the formation of cancer. However, in the tumor stage, it is crucial to enhance anti-tumor immunity in order to successfully suppress tumor growth. The TME can be enhanced by optimizing the recruitment of immune cells through metabolic modulation. The metabolites produced by tumor cells and immune cells have an impact on the activation and phenotypic changes of immune cells, as well as the regulation of immunological checkpoints. The novel study methodology can be targeted towards various metabolic pathways.

In this review, we provide a framework to introduce the inducing factors of the development of CAC and summarize the major agents targeting the pathways during all the stages of CAC associated with immunometabolism. The summary of immunometabolism in each stage of CAC and targeted medications offer possibilities for treating and overcoming CAC.

## 2 Colitis associated colorectal cancer

### 2.1 Relationship between colorectal cancer and colitis associated colorectal cancer

Comparable to sporadic colorectal cancer, CAC is characterized by clonal expansion of somatic cells and genetic modification. A dysplastic polyp is frequently observed in sporadic colorectal cancer lesions; it is discrete, generally visible, and few in number. Nevertheless, CAC is distinguished by extensive areas of persistently inflamed mucosa that are susceptible to metastatic transformation, a process known as “field cancerization,” which complicates endoscopic detection and resection (Ullman et al., 2009; Shah and Itzkowitz, 2022). Loss of APC and KRAS mutations transpire during the initial stages of sporadic CRC development, whereas these alterations transpire towards the latter stages of CAC progression. Furthermore, it is worth noting that mutations of p53, SMAD4, and DCC occur relatively late in sporadic CRC, whereas they are relatively early in CAC. Prognosis and mortality are typically worse for patients with CAC compared to CRC patients without a prior IBD history. This may be due to the difficulty in detecting premalignant lesions in CAC at an early stage (Brackmann et al., 2010; Adams et al., 2013; Renz et al., 2013; Fantini and Guadagni, 2021).

### 2.2 Epidemiology of colitis associated colorectal cancer

The risk of CRC is approximately two to three times higher in patients with inflammatory bowel disease. The severity, duration, and extent of colitis, all of which contribute to an increased extent of oxidative DNA damage, are also associated with an increased risk of CRC (Kawanishi et al., 2006). Recent meta-analysis indicates that the incidence of IBD-associated cancer is 0.1%, 2.9%, and 6.7% over 10, 20, and 30 years, respectively, after the advent of UC (Choi et al., 2015). In contrast to UC, a recent investigation has demonstrated a progressive increase in the incidence of CRC by 2.9%, 5.6%, and 8.3% over a span of 10, 20, and 30 years, respectively, following the onset of CD (Canavan et al., 2006). In addition, primary sclerosing cholangitis, male sex, and immediate family with CAC verified risk factors for CAC, which will increase the incidence of CAC (Birch et al., 2022).

### 2.3 Therapy for colitis associated colorectal cancer

Treatment for CRC involves surgical removal of the tumor, radiation therapy, chemotherapy, and the use of biologic medicines. Upon the detection of a malignant tumor, it is advisable to explore the option of total colectomy.

Nevertheless, numerous unresolved difficulties persist. Drug resistance is typically unavoidable in a significant number of individuals with CAC. Further research is needed to investigate the utilization of anti-inflammatory drugs while preserving anti-tumor immunity. This research should focus on inhibiting T cells and macrophages, suppressing the production of immunosuppressive cytokines by immune cells, and preventing the recruitment of MDSCs. These factors have been found to contribute to the ineffectiveness of anti-cancer treatments. Metastasis of tumors in CRC poses a challenge as current medicines have little effectiveness (Gorlick and Bertino, 1999; Longley et al., 2006; Spranger et al., 2015; O’Donnell et al., 2016; Zaretsky et al., 2016).

Key approaches to decrease the occurrence of CAC involve timely identification of IBD, ongoing monitoring using endoscopy, and management of persistent inflammation (Bezzio et al., 2017). Nowadays, Mesalamine is the only known substance that has been proven to decrease CRC in individuals with IBD. Mesalamine works by acting as a specific activator of PPAR-γ and as an inhibitor of PAK1 and NF-kB (Shun-Wen et al., 2022).

Hence, in light of the limited effectiveness and diverse adverse reactions associated with current treatments, it is imperative to explore the development of alternative medicines that might safeguard against both the first inflammatory phase and the subsequent tumor stage (Table 1).

**TABLE 1:** Summary of herbal medicine and agents targeted metabolism.

Drug/intervention	Stage	Mechanism
Dihydroartemisinin^[152, 153]^	Chronic inflammation	Inhibit macrophage-related inflammatory response
	Carcinoma	Inhibit the growth of tumor cells
Arctigenin^[155]^ and Atractylenolide^[156]^	Chronic inflammation	Promote the production of inflammatory cytokines and downregulate FAO in macrophages
Berberine^[157, 158]^	Carcinoma	Inhibit the growth of tumor cells and regulate intestinal microorganism
Dimethyl itconate^[159]^	Chronic inflammation	Inhibit the formation of immunosupressive TME
Glycolysis inhibition^[160]^	Carcinoma	Inhibit the metabolism of tumor cells
Inhibition of glutamine intake^[161]^	Carcinoma	Inhibit the metabolism of tumor cells
IDO inhibitor^[97]^	Carcinoma	Inhibit the formation of immunosupressive TME and restore anti-tumor immunity

Abbreviation: FAO, arctigenin downregulate fattyacidoxidation; TME, tumor microenvironment; IDO, indoleamine 2,3¬ dioxygenase.

## 3 Immunometabolism and colitis associated colorectal cancer

### 3.1 Immunometabolism

Immunometabolism is the growing area that focuses on the interaction between metabolism and immunity, both at the systemic and cellular levels.

Immune cells are influenced by internal metabolism at multiple levels. Metabolic adaptation refers to the reciprocal regulation of immune cell metabolism and activities. Immune cells undergo differentiation into distinct subsets based on their rapid metabolic reprogramming, which involves processes such as glycolysis, the tricarboxylic acid (TCA) cycle, the pentose phosphate pathway, fatty acid oxidation, fatty acid synthesis, and amino acid metabolism (O’Neill et al., 2016).

However, there is a growing belief that the innate and adaptive immune system have a role not just in autoimmune diseases, but also in non-immune disorders such as Metabolic syndrome, cardiovascular diseases, neurodegenerative diseases, and malignancies.

### 3.2 Diseases associated immunometabolism

The underlying cause of metabolic syndrome is chronic inflammation triggered by adipose tissue, in which cytokines and immune cells play a crucial role. Saturated fatty acids from adipose tissue cause the release of pro-inflammatory cytokines through the TLR4 of macrophages, which in turn increase the disintegration of adipose tissue, generating a vicious loop of inflammation (Hachiya et al., 2022). Nevertheless, certain studies have indicated that TLR4 does not function as a receptor for saturated fatty acids. However, it does play a crucial role in the inflammation that is triggered by these fatty acids. Activation of TLR4 promotes the process of aerobic glycolysis, restricts the tricarboxylic acid cycle (TCA), and hinders the oxidation of fatty acids in macrophages (Lancaster et al., 2018).

Alzheimer’s disease (AD) is the prevailing neurodegenerative condition. Mounting evidences indicate that immune cells in AD patients undergo a range of metabolic alterations. Microglial responses to AD pathologies play primary and crucial roles in the progression of AD. The dysfunction of microglia in advanced Alzheimer’s disease (AD) may be linked to compromised glucose metabolism. Furthermore, amyloid-β triggers the activation of the NLRP3 inflammasome in microglia by stimulating Syk and suppressing adenosine monophosphate–activated protein kinase (AMPK) (Jung et al., 2022). Deactivated AMPK induces metabolic dysregulation, mitochondrial fragmentation, and reactive oxygen species formation (Lee et al., 2022). Mitochondria dysfunction results in increased oxidative stress, impaired mitochondrial respiration, and suppressed mitophagy (Rubio-Araiz et al., 2018; Bennett and Liddelow, 2019; Butterfield and Halliwell, 2019).

Autoimmune disorders (AIDs) are defined by abnormal immune responses and the corresponding signaling pathways that regulate cell development, death, and survival. Researchers have observed that immune cells’ metabolic abnormalities may have significant implications in the progression of AIDS (Yang et al., 2015), such as rheumatoid arthritis and systemic lupus erythematosus. Hence, the suppression of aberrant metabolic pathway could potentially be employed as a therapeutic approach for the treatment of AIDS (Warner et al., 1994; Gu et al., 2016; Wang et al., 2021).

Tumor cells frequently transition to aerobic glycolysis for energy production in the TME, a phenomenon that has also been observed in the presence of inflammation. Thus, it is possible to hypothesize that metabolic transformation occurs not only during the progression from chronic inflammation to tumorigenesis, but also in the later stages of CAC. Hypoxia does indeed exist in the environment of both inflammation and tumors (DeBerardinis and Chandel, 2016; Dingding et al., 2017).

The TME, which help the cancer cells escape from the killing of immune cells, and inflammation environment and multiple pro-inflammtion cytokines regulate the gene expression and promotes them to reprogram metabolism (Zhang et al., 2019). Despite sharing the same environment, there are significant differences in the metabolic reprogramming between immune cells and tumor cells. These differences allow us to identify the characteristics of immunometabolism and identify suitable targets for CAC (Leone and Powell, 2020). Hence, we can infer that immunometabolism is linked to CAC.

## 4 Immunometabolism in the development of colitis associated colorectal cancer

**FIGURE 1:**
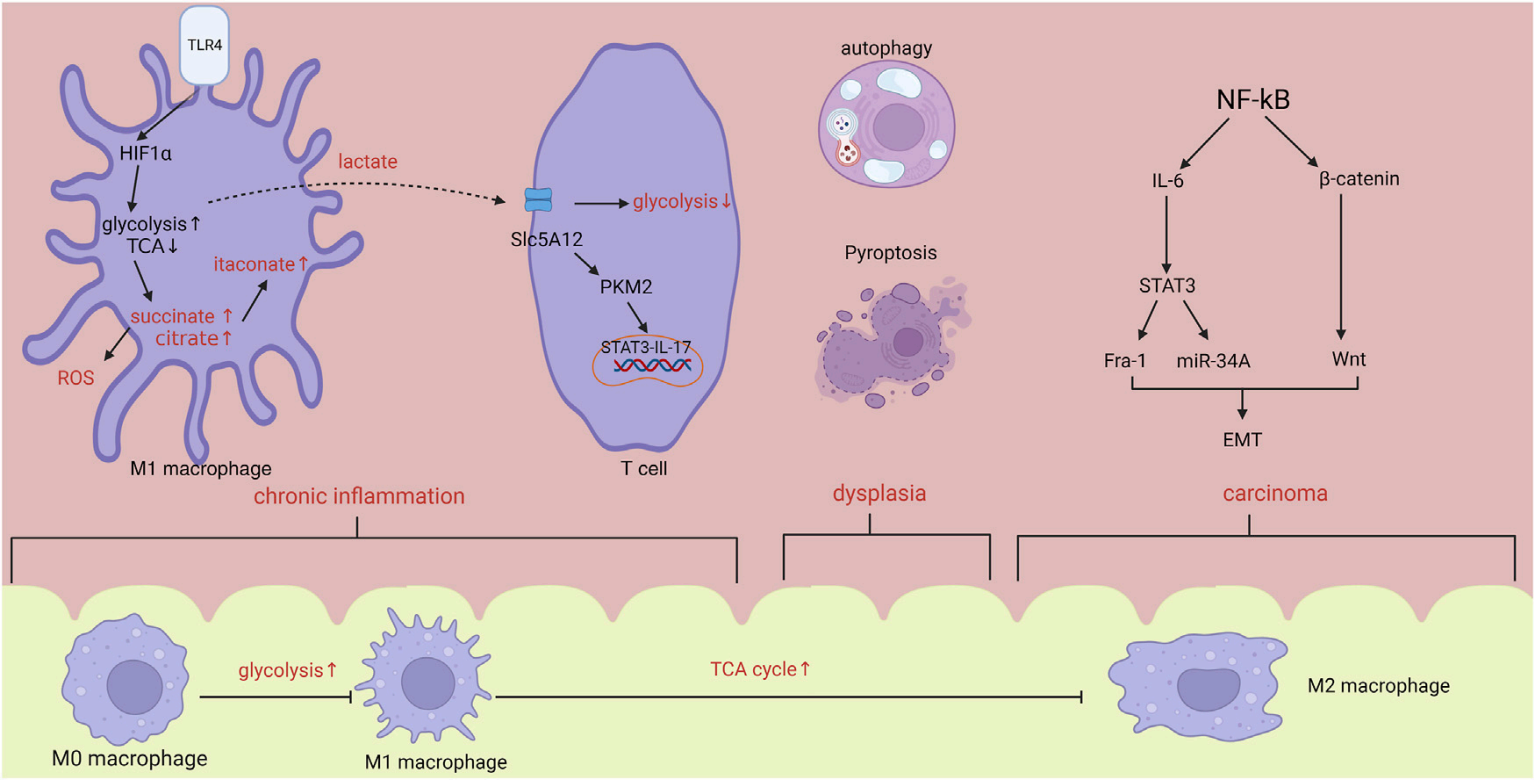
In the stage of chronic inflammation, hypoxia induced HIF1α increase will promote the activation of glycolysis and inhibition of TCA cycle, leading to the accumulation of citrate, succinate and itaconate. Lactate synthesized from glycolysis will activate T cells via PKM2/STAT3/IL-17 pathway. Within tumor microenvironment, activated NF-kB will accelerate the progression of metastasis by IL-6 and β-catenin/Wnt pathway. From inflammation to carcinoma, body changes from pro-inflammatory state to anti-inflammatory state. Abbreviations: HIF1α, hypoxia inducible factor-1α; ROS, reactive oxygen species; EMT, epithelial-mesenchymal transformation.

The term “dysplasia-carcinoma sequence” is employed to characterize the unpredictable progression of CAC (Baker et al., 2018). CAC arise as a result of prolonged inflammation and progress through a series of stages, starting with indeterminate dysplasia, followed by low-grade dysplasia, high-grade dysplasia, and ultimately leading to carcinoma (Ullman and Itzkowitz, 2011). The etiology of CAC remains incompletely elucidated, and it is commonly attributed to a combination of variables including inflammatory response, immunological microenvironment, and disturbances in intestinal flora. This work aims to elucidate the relationship between the development of CAC and immunometabolism in the context of chronic inflammation, cellular apoptosis and proliferation, TME creation, as well as tumor invasion and metastasis (Figure 1).

### 4.1 Chronic inflammation

The initial phase of CAC is characterized by persistent inflammation, as evidenced by the effective utilization of anti-inflammatory medications to prevent CAC (Lasry et al., 2016). Chronic inflammation results from the activation of signaling pathways without being stimulated by injure (Hirano et al., 2020) and often contribute to continuous tissue damage, accumulation of immune cells, fibrosis and the development of cancer, including cellular transformation, survival, proliferation, invasion, angiogenesis and metastasis (Hanahan and Weinberg, 2011; Lasry et al., 2016).

#### 4.1.1 Metabolic reprogramming in chronic inflammation

Immune cells undergo metabolic programming and use metabolic pathways to control cell fate. During IBD, the difference between oxygen supply and exhaustion in the mucosal renders the inflamed regions severely hypoxic (Van Welden et al., 2017).

Macrophages play a crucial role in maintaining the balance and stability of the intestines and serve as guardians of the immune system in the intestines. Macrophages experience metabolic and functional alterations as a result of the microenvironment’s effect. Following the interaction between lipopolysaccharides (LPS) derived from gut microbiota and Toll-like receptor 4 (TLR4), hypoxia-inducible factor 1-alpha (HIF1-α) is consistently activated. This activation can be further intensified by nuclear factor kappa B (NF-kB) and facilitates the metabolic reprogramming of macrophages (Corcoran and O’Neill, 2016), manifesting as the increase of glycolysis and pentose phosphate pathway (PPP), and break in TCA, which render the accumulation of citrate, succinate and lactate.

The immune response gene (IRG1) synthesizes the bioactive metabolite itaconate from citrate. Itaconate has the ability to inhibit the action of succinate dehydrogenase (SDH), hence decreasing the formation of ROS (Lampropoulou et al., 2016; Fremder et al., 2021). Research has shown that itaconate has the ability to hinder the process of glycolysis in M1 macrophages and the TCA in M2 macrophages. As a result, this leads to a decrease in the buildup of macrophages and MDSC (Runtsch et al., 2022), which play an important role in immunosuppressive environment. However, some researchers also found that itconate can downregulate the expression of PPARγ, an inhibitor of CAC, and promote the anti-inflammatory cytokines from M2 macrophages (Scheurlen et al., 2022), all of which can promote the development of CAC. therefore, itaconate could potentially be utilized as a preventative agent against tumorigenesis. Moreover, its pro-cancer properties should be taken into account. Succinate can be oxidized by succinate dehydrogenase thus increasing the reactive oxygen species (ROS) from mitochondria and stabilizing the transcriptional factor HIF1α, which in turn promotes the proinflammatory transformation of macrophages (Corcoran and O’Neill, 2016).

Excessive activation of glycolysis can enhance the synthesis of lactate, which can be absorbed by CD4+T cells and CD8+T cells using the lactate transporters SLC5A12 and SLC16A1, respectively. Elevated levels of lactate can hinder the glycolysis process triggered by the interaction between chemokine CXCL10 and chemokine receptor CXCR3. This restriction confines CD4+T cells to inflamed regions and triggers the phosphorylation of STAT3 by causing the movement of pyruvate kinase M2 (PKM2) into the nucleus. Consequently, this leads to an increase in the production of IL-17 in T cells and proglycolytic enzymes in macrophages (Luo et al., 2011; Haas et al., 2015; Pucino et al., 2019).

Consequently, in response to the extremely low oxygen levels, immune cells undergo metabolic reprogramming and generate different metabolites, which subsequently control the function of immune cells themselves.

#### 4.1.2 Immune response in chronic inflammation

Macrophages and lymphocytes exacerbate the mucosal damage induced by inflammation and facilitate the transition from inflammation to tumor development. Lymphocyte proliferation and dysregulation, along with the disruption of pro-inflammatory cytokines and anti-inflammatory mediators, are the primary contributors to intestinal inflammation. These cytokines stimulate the growth, survival, and spread of cells in the lining of the intestines by activating specific transcription factors, namely, STAT1, STAT3, and NF-kB (Zhang et al., 2023). The presence of CAC is not just determined by the presence or severity of inflammation, but rather by the specific type of inflammatory response. The inflammatory response, mainly mediated by F4/80+Ly6Chigh macrophages, is related to the initiation of CAC. Macrophage-associated cytokines IL-1 β, TNF- α and IL-6 increase more in tumor-associated colitis and promote the stemness of Dclk1+ tuft cells, which is the cellular origin of colon tumors (Frigerio et al., 2021).

NF-κB has a critical role in regulating the connection between inflammatory responses and the development of cancer. Upon stimulation of TLR4, NF-kB disassociates from I-kB and translocates into the nucleus to bind with certain genes that are involved in the synthesis of proinflammatory cytokines and carcinogenesis, including COX-2, inducible nitric oxide synthase (iNOS), and vascular endothelial growth factor (VEGF) (Porter et al., 2021). Damage-associated molecular patterns S100A8 and S100A9 play an important role in colitis by binding to their pattern recognition receptors PRR, especially TLR4. rCT-S100A8/9 that consisted of TLR4-and RAGE-inhibiting motifs derived from S100A8 and S100A9, and conjugated with a CT peptide (TWYKIAFQRNRK) for colon-specific delivery, inhibits the production of NLRP3 inflammatory bodies mediated by NF-kB by inhibiting the binding of S100A8 and S100A9 to TLR4. The interaction between NLRP3 and ASC can promote the maturation of IL-1 β and IL-18 (Riddell et al., 1983). The upregulation of TGF-β can not only inhibit the growth of benign or low-grade cancer, but also stimulate the progression of malignant and metastatic tumors. TGF- β not only inhibits tumor by maintaining epithelial homeostasis and regulating cell growth, cell cycle, differentiation and apoptosis, but also plays an immunosuppressive role by affecting immune cells. SMAD4, as a signal transduction molecule in the downstream signal transduction pathway of TGF- β, can inhibit the role of pro-inflammatory factors in colonic epithelium. Specific knockout will increase the chemoattraction of CCL20 to CCR6+ immune cells, thus increasing the susceptibility to the development of CAC. However, TGF- β promotes the development of IL-17+IL-22 + T cells, and the produced IL-22 can regulate the tumor niche and promote the occurrence of CAC by activating transcription factor STAT3 to promote the gene expression of core stem cells. SMAD4 knockout can increase the expression of IFN- γ in T cells. IFN- γ promotes epithelial cell transformation by inhibiting the production of tumor suppressor 15-hydroxyprostaglandin dehydrogenase (15-PGDH) in colonic epithelial cells, thus accelerating the occurrence of gastrointestinal epithelial malignant tumors, and tissue resident CD4^+^ effector memory T cells enhance the production of pro-inflammatory mediators, both of which are regulated by TGF-β (Nguyen et al., 2014; Lazarou et al., 2015; Packiriswamy et al., 2017).

### 4.2 Dysplasia

Low-grade dysplasia occurs when the mucosa, which has been genetically modified due to long-term inflammation, is more likely to develop into cancer (Devenport et al., 2021). Colorectal dysplasia can be defined as a neoplastic alteration of the intestinal epithelium that remains confined within the basal membrane (Deretic, 2021).

#### 4.2.1 Cells death

Cell death is a crucial mechanism for maintaining homeostasis in the body, encompassing autophagy, ferroptosis, pyroptosis, and apoptosis.

Autophagy facilitates the removal of faulty organelles, membranes, and proteins by means of lysosomal degradation, hence playing a crucial role in the proper growth and specialization of immune cells. There is a strong correlation between autophagy, inflammation, and the metabolic processes of immune cells in CAC. ROS generated during persistent inflammation can trigger autophagy in the intestines, whereas autophagy mitigates ROS levels through mitophagy, so suppressing intestinal inflammation (Devenport and Shah, 2019). Thus, ATG5 and ATG16L1-mediated epithelial autophagy eliminates toxicogenic microbacteria and inflammatory cytokines, preventing oxidative damage-induced carcinogenesis (Koppula et al., 2021; Kim et al., 2022). In the hypoxic tumor microenvironment, epthelial autophagy activates the AMPK/ULK1 and JaK2/STAT3 pathways and sustains mitochondrial metabolism to nourish cells and promote cell proliferation. As the primary regulator of cellular metabolism, AMPK controls autophagy and immunometabolism, affecting immune cell activity. It also promotes TME production through anti-inflammation and suppressive immune response (Chen et al., 2020; Xu et al., 2020). Recently, researchers discovered that a special transcriptional factor, Small leucine zipper protein (sLZIP), can provide tumor cells with glutamine for ATP synthesis, induce autophagy to promote tumor growth, and activate the NrF2 pathway to maintain redox stability and reduce ROS. SLZIP may prevent ferroptosis by increasing NrF transcription, which regulates SLC7A11 expression (Jiang et al., 2015; Tan et al., 2020; Zhang et al., 2020). In summary, autophagy in the intestinal epithelium hinders the onset of cancer but facilitates the growth and advancement of colon cancer.

Ferroptosis is an iron-dependent cell death caused by ROS-induced lipidperoxidation. Ferroptosis plays a paradoxical role in intestinal disorders, as blocking it reduces colitis symptoms but promotes colon carcinogenesis (Karin, 2006; Dan et al., 2008). P53 is a kind of tumor suppressive gene, which serves as an important role in preventing from cancer development by mediating cell-cycle arrest, senescence and apoptosis. Another research found that, P53 can inhibits the expression of the cystine/glutamate transporter (Xc-system) subunit SLC7A11, thereby suppressing cystine uptake, biosynthesis of glutathione, and in turn increasing the incidence of ferroptosis (Greten et al., 2004). Pyroptosis is an inflammatory form of programmed cell death that is triggered by the activation of caspase-1 through inflammasomes. This activation leads to the release of pro-inflammatory cytokines, including IL-1β, as well as the cleavage of gasderminD into gasdermin-N and gasdermin-D fragments. These fragments then puncture the cell membrane, causing the release of various damage-associated molecular patterns (DAMPs). The promotion of colon cell proliferation via the activation of the mitotic signalling pathway ERK1/2 by the high mobility group box-1 protein, which is generated via GSDME-mediated pyroptosis (Grivennikov et al., 2009). In contrast, after indentifying cancer cells, natural killer cells and cytotoxic T lymphocytes (CTLs) release large amounts of perforin and granzymes, which can cleave gasdermin-E, leading to pyroptosis of tumor cells (Pagano et al., 2022). Hence, ferroptosis and pyroptosis can have both beneficial and detrimental effects during tumour progression. Further research is needed to investigate how to effectively control the timing and intensity of CAC treatment.

#### 4.2.2 Tumor proliferation

Persistent proliferation signals and suppressed apoptosis promote more cells to enter the cell cycle, which initiates the tumorigenesis. The signaling via mechanistic target of rapamycin (mTOR), such as PI3K-AKT-mTOR, which makes NF-κB an important endogenous tumor promoter is involved in tumor proliferation and survival. mTOR serves as the catalytic centre for two distinct complexes known as mTORC1 and mTORC2. mTORC1 integrates signals from growth hormones and nutrients to regulate processes including as protein synthesis, growth, autophagy, and ribosome biogenesis. These functions can be suppressed by AMPK (Ku et al., 2015). NF-kB-induced IL-6 stimulates STAT3 activation in intestinal epithelial cells and promotes tumorigenesis in CAC through the inhibition of apoptosis and induction of cell proliferation (Azoitei et al., 2016; Siska et al., 2017), although IL-6 does not have significant effect on early tumor promotion in the CAC. GPR35 on the cell membrane of tumor associated macrophages (TAM) play a vital role in the tumor angiogenesis by activating Na/K ATPase and its downstream signal src kinase to promote the release of angiogenic cytokines such as VEGF and IL-8, which involve in the growth and progression of CAC (Warburg, 1956a; Warburg, 1956b). Furthermore, PKM2 and the p65 component of NF-kB migrate to the nucleus and stimulate the production of VEGF, so facilitating the development of blood vessels (Ruiz-Iglesias and Mañes, 2021).

### 4.3 Carcinoma

#### 4.3.1 Tumor microenvironment and immunometabolism

Chronic inflammation can contribute to the development of an immunosuppressive TME, resulting from metabolites secreted by tumor and immune cells, in which cancer cells thrive and compete with immue cells for the limited glucosis (Porter et al., 2021). TME can in turn alter the metabolism of T cells and macrophages to impair anti-tumor immunity (Liu et al., 2017). The Warburg effect, a well-documented metabolic reprogramming process, signifies a metabolic transition in cancer cells from aerobic mitochondrial oxidation to glycolysis in order to supply the energy required for accelerated cell proliferation (Hao et al., 2012; Ip et al., 2017), which is a master feature of tumor cells and leads to the accumulation of lactate in TME. Tumor cells can downregulate the pyruvate carrier of mitochondrial which can transfer pyruvate from cytosol to mitochondrial, leading to the accumulation of pyruvate. Then it will be converted into lactate and become the fuel of cells in TME (Italiani and Boraschi, 2014). Extracellular lactate can alter the metabolism of immune cells, stimulate the signal pathway in immune cells, and decrease the extracellular PH value, all of which influence the function of immune cells in the TME.

Macrophages, apart from infiltration and secretion of cytokines, disrupt the equilibrium of polarization and exhibit diverse phenotypes in response to distinct environmental stimuli. A deficiency in glucose and amino acids within the TME will restrict macrophage metabolism, resulting in decreased production of NADPH, ROS, and succinate, all of which inhibit the function of M1-type macrophages. M2 macrophages, in contrast to M1 macrophages, possess a functional TCA cycle, adequate OXPHOS with glutamine and fatty acids as fuels, and a notably high expression of arginase-1 to deplete arginine and generate polyamine, thereby potentially impeding the immune response. α-ketoglutarate (a-KG), which is generated through the breakdown of glutamine, serves as a metabolic reprogramming checkpoint for the polarization of M2 type macrophages. It facilitates the consumption and oxidation of fatty acids (Finlay et al., 2012). The solute carrier family 7 member 2 (SLC7A2) is responsible for transporting L-arginine. Recently, a number of researchers discovered that SLC7A2-deficient rodents had more polarized M2 macrophages, which increased the risk of CAC. Consequently, L-Arg supplementation may decrease the long-term risk of colorectal cancer. Anti-inflammatory cytokine production, such as IL-10, inhibits glycolysis and M2 polarisation of macrophages via the IL10/STAT3 pathway, which can impede mTORC1 (Cham et al., 2008).

CD8^+^T cells plays an important role in antitumor response and their responses become impaired during tumor progression. In TME characterized by hypoxia, the upregulated transcriptional activity of MYC and HIF-1 will activate the expression of glycolytic enzyme (Angelin et al., 2017). Nevertheless, the insufficiency of glucose will impact the glycolysis process, subsequently mitigating the mTOR signalling pathway. This, in turn, may stimulate the differentiation of regulatory T cells (Tregs) and impede the functionality of functional T cells (Yang et al., 2016). Treg cells prefer TCA cycle and mitochondrial respiration but have high flexibility in metabolism and can thrive in TME by metabolic reprogramming, which is enriched with low glucose and high lactate (Sinclair et al., 2013). The expression of STAT3 in cancer-associated fibroblasts promotes the recruitment of myeloid-derived suppressor cells (MDSCs) in to the tumor by the production of C-C motif chemokine ligand 2 (CCL2) to create an immunosuppressive TME. Some studies have shown that granulocytic MDSCs (G-MDSC) from peripheral blood of patients with IBD can enhance autologous T cell proliferation and exocrine secretion, and promote the differentiation of Monocytic MDSCs (M-MDSC) into M2 macrophages by down-regulating STAT3 activity by miR935p (Geiger et al., 2016; Liu et al., 2018). Additionally, amino acid metabolism is critical for immunometabolism. Leucine is required for both the mTOR signaling pathway and the differentiation of T cells (Schwitalla et al., 2013). Arginine is swiftly utilised by activated T cells, which enhances their capacity to eliminate tumour cells (Liu et al., 2015). Tryptophan regulates the immune system. The consumption of tryptophan activates the stress-response kinase GCN2 of immune cells, which reduces the proliferation of T cells and increases the differentiation of Tregs, resulting in immune tolerance to tumor cells. However, the ability of colon-specific indoleamine 2,3- dioxygenase (IDO) to inhibit effector T cells does not depend on the metabolic depletion of tryptophan. The inhibitory effect of GCN2 is more obvious in antigen presenting cells. Under the stimulation of a variety of cytokines, the expression of IDO in tumor cells increases, which catalyzes the cleavage of tryptophan, which leads to the depletion of tryptophan and the production of immunomodulatory molecule, Kynurenine (Kyn). Kyn promoted AhR translocation also increased the transcription of Foxp3, a marker of Treg in CD4+T cells, thus inhibiting the attack of CTL on tumor cells (Rokavec et al., 2014).

The establishment of the TME is crucial for the growth and spread of tumour cells, as it generates an immunosuppressive setting that allows tumour cells to evade immune system detection.

#### 4.3.2 Epithelial–mesenchymal transition

Studies indicate that around one-third of patients diagnosed with CRC will experience the development of metastases, with over 75% of these cases specifically involving liver metastasis. The TME can exert effect on many critical stages of metastasis. Epithelial to mesenchymal transition (EMT) refers to the phenomenon when epithelial cells undergo a separation from their adjacent cells and acquire the migratory properties of stromal cells.

The process of EMT plays a crucial role in the progression of cancer, from its early stages to the later stages of invasion and metastasis. During this process, there is a drop in the levels of E-cadherin and an increase in the levels of N-cadherin. The activation of NF-kB enhances the Wnt signalling pathway through the interaction of RelA/p65 with β-catenin, resulting in the dedifferentiation of intestinal epithelial cells (Sun et al., 2016). Furthermore, NF-kB stimulates the synthesis of IL-6 in bone marrow cells, leading to the persistent activation of STAT3. This, in turn, enhances the expression of fos-related antigen-1 (Fra-1), which is closely linked to the spread of cancer and a poor prognosis (Nan et al., 2021). The tumor suppressor p53 can trigger the activation of miR-34, which in turn can impede the process of EMT. Researchers discovered that the process of EMT and invasion, caused by IL-6, necessitates the inhibition of miR-34A. Additionally, the spread of tumors is influenced by the IL-6/STAT3/miR-34A pathway (Fensterheim et al., 2018). HIF1 α has the ability to stimulate the activation of two transcription factors, Snail and Twist, which play a role in controlling E-cadherin. This activation leads to the promotion of tumour invasion and resistance to chemotherapy (Yang et al., 2020). Tumor cells alter their metabolic pathways, such as increasing glycolysis and fatty acid oxidation while suppressing oxidative phosphorylation, in order to fulfil their bioenergy requirements associated with EMT (Jiang et al., 2023).

Anyway, the occurrence of EMT involves various transcription factors and signal pathways, forming a complex network, which plays an important role in the metastasis and invasion of CAC, and is closely related to chemotherapy resistance.

## 5 The role of microbiota

Gut microbiota is involved in the development of CAC. They influence the pretumor environment, including mediating DNA damage and inducing carcinogenic signaling pathways, and promote the formation of TME, resulting in the infiltration of immune cells and condition of suppressive immune response.

The chemicals produced by the microbiota within the pre-tumor microenvironment can contribute to the development of chronic inflammation. The microbiota-derived lipopolysaccharides (LPS) stimulate the accumulation of macrophages with monocyte-like characteristics by binding to Toll-like receptor 4 (TLR4) and triggering the expression of hypoxia-inducible factor 1-alpha (HIF-1α). This, in turn, stimulates aerobic glycolysis and ultimately results in the polarization of macrophages (Scott et al., 2018; Shao et al., 2021).

A recent study has shown that Akkermansia muciniphila has a positive effect on reducing CAC by encouraging metabolic reprogramming in the TME through the action of Amuc_2172, a specific type of acetyltransferase. This acetyltransferase induces the secretion of heat-shock protein 70, which in turn enhances the activation of CTL to effectively eliminate tumour cells. Researchers have discovered a potential link between microorganisms and CAC in immunometabolism (Schulthess et al., 2019). On the contrary, pathogenic bacteria include *Bacteroides fragilis*, *Fusobacterium* nucleatum, *Enterococcus faecalis*, Colibactin-producing *Escherichia coli*, *etc.*

The presence of *Bacteroides fragilis* in the fecal samples of CAC patients decreased dramatically, as shown by data. The utilization of wide-ranging antibiotics results in dysbiosis of the intestinal microbiota and expedites the advancement of colon cancer. Nevertheless, following gavage transplantation with *Bacteroides fragilis*, a bacterium capable of generating short-chain fatty acids (SCFAs), the intestine can counteract the impact of BSAB. SCFAs, particularly butyrate, can subsequently suppress the NLRP3 signaling pathway, so inhibiting the release of proinflammatory mediators like IL-18 and IL-1β. This, in turn, reduces intestinal inflammation and restricts the development of CAC (Geis et al., 2015). Furthermore, butyrate, acting as an inhibitor of HDAC3, has the ability to decrease glycolysis while stimulating OXPHOS and lipid metabolism in macrophages. This, in turn, enhances their polarisation towards the M2 phenotype (De Simone et al., 2013; Cao et al., 2021). Thus, *Bacteroides fragilis* can inhibit the onset of CAC but facilitate the subsequent formation of colon tumors. Researchers discovered that enterotoxigenic *Bacteroides fragilis* (ETBF) stimulates the development of Th17 cells by reducing the expression of miR-149-3p, leading to the infiltration of Th17 cells into the colon (Rubinstein et al., 2013; Hu et al., 2021), and finally contributes to the evolvement from chronic inflammation to tumor (Gur et al., 2015).

*Fusobacterium* nucleatum (Fn) induces the polarization of macrophages into the M2 phenotype by activating the TLR4/NF-kB/S100A9 pathway. It also promotes the infiltration of other myeloid cells through the action of its metabolites. Fn utilizes its Fap2 adhesin to hinder the activity of natural killer cells and ultimately suppresses the immune response against tumors. These findings collectively suggest that Fn plays a role in the development of the TME (Wang et al., 2008). Besides, Fn can stimulate tumor cells proliferation and angiogenesis by activating the Wnt/β-catenin pathway and producing NF-kB (Cuevas-Ramos et al., 2010; Wang et al., 2012). *E. faecalis* and Colibactin-producing *E. coli* can cause DNA damage in the epithelium by triggering elevated amounts of ROS, resulting in genomic instability and mutation (Axelrad et al., 2016).

The interaction between the immune system and gut microbiota is crucial for the onset and progression of CAC.

## 6 Therapy for colitis associated colorectal cancer

### 6.1 Anti-inflammation drugs

CAC is a biological process characterized by the initial occurrence of chronic inflammation followed by the formation of tumors. Monotherapy with anti-inflammatory medications is unlikely to be highly beneficial for individuals with CRC. However, these drugs may be utilized, either alone or in combination with other techniques, for the purpose of preventing carcinogenesis (Bos et al., 2006; Terzić et al., 2010) (Table 2).

**TABLE 2:** Summary of potential anti-inflammation drugs in CAC.

Target	Drug/Intervention	Anti-tumor effect
COX enzymes	Aspirin/AT-SPMs	Down-regulating PD-1 in macrophages and CD8+T cells to enhance anti-tumor immunity
cytokines	TNF-α inhibitor (infliximab)	Inhibit CAC growth
	Blocking IL-17A	Enhance the role of PD-1 blockers and suppress the formation of TME
IL-6/STAT3	Combined use of anti-IL-6R antibody and STAT3 inhibitor (JSI-124)	Inhibit the occurrence of CAC
TLR4	TLR4 inhibitor (TAK-242)	Prevent macrophage infiltration
	rCT-S100A8/9	Block the S100-PRR interaction
NF-kB	HDAC inhibitor (TMP195)	Decrease the activation of NF-kb, promote the M1 macrophages polarization and enhance the role of PD-1 blockers
	PI3K3R3 inhibitor TAT-N15	Decrease the activation of NF-kb and improve the intestinal integrity

Abbreviations: AT-SPMs, aspirin-triggered specialized proresolving mediators; PD-1, programmed cell death protein-1; CAC, colitis associated colorectal cancer; TME, tumor microenvironment; HDAC, histone deacetylase; PIK3R3, phosphoinositide-3-kinase regulatory subunit 3.

#### 6.1.1 5-Aminosalicylates

5-Aminosalicylates (5-ASA) can reduce the risk of CAC, which may be related to its anti-inflammatory effect. 5-ASA can inhibit tumor growth by affecting multiple molecular pathways. For example, it reducing the β-catenin accumulation in APC-mutated cells and reduce the occurrence of DNA mutation. However, A population-based study showed that 5-ASA does not reduce the risk of CAC (Lanas, 2009; Campregher et al., 2010; De Matteis et al., 2022). Hence, it is worth contemplating the potential of utilizing 5-ASA for the prevention of CAC.

#### 6.1.2 Nonsteroidal anti-inflammatory drugs

Aspirin is commonly recognised as a chemopreventive medication that lowers the risk of CRC by inhibiting COX enzymes. This inhibition leads to a decrease in the production of prostaglandins and thromboxane, which are known to be associated with the development and spread of colon tumours (Din et al., 2012). In addition, aspirin induced acetylated COX-2 promotes the production of aspirin-triggered specialized proresolving mediators (AT-SPMs), including AT-lipoxin A4 and AT-resolvin D1, which can downregulate the expression of programmed cell death protein-1 (PD-1) in macrophages and CD8^+^T cells, and regulate tissue metabolism, reducing the production of several glycolysis intermediates, thereby improving anti-tumor immunity and reducing the incidence of drug resistance. Besides, aspirin also decreased the expression of IL-17A in CD4^+^ T cells and the abundance of Tregs in colon tissue. These T cell subsets are associated with supporting angiogenesis, tissue glycolysis and immunosuppression, respectively (Popivanova et al., 2008). Therefore, AT- AT-SPMs may be a potential drug in the treatment of colon tumors.

#### 6.1.3 Agents targeted cytokines

The secretion of cytokines by immune cells plays a crucial role in the entire process of CAC development. Therefore, inhibiting tumor-promoting cytokines could potentially serve as a therapy and preventive approach.

TNF-α, a chronic inflammatory mediator, facilitates the onset and progression of CAC. TNF-α antagonists have the ability to impede the establishment of CAC in mice that have been treated with dextran sodium sulphate (DSS). Additionally, the number of CAC cells dropped dramatically in animals that lack the TNFR1. Consider initiating early infliximab therapy in high-risk patients with IBD to proactively prevent the eventual onset of colon cancer (Kim et al., 2010). Besides, anti-human IL-6R (tocilizumab), IL-17 inhibitors (brodalumab, secukinumab, and ixekizumab) and IL-12/23 inhibitors (ustekinumab) have been found to be the most safe and effective in the treatment of autoimmune diseases. Recent studies have identified IL-17A as a potential target for therapy. IL-17A has been reported to enhance the recruitment of MDSCs and boost the immunosuppressive activity of Treg cells. Additionally, IL-17A can raise the expression of PD-L1 through the p65/NRF1/miR-15b-5p pathway, leading to resistance against PD-1 therapy. Additionally, the inhibition of IL-17A also enhanced the effectiveness of anti-PD-1 treatment in a mouse model of microsatellite stable (MSS) colon tumor (Wang et al., 2013; Liu et al., 2021).

At present, it is generally believed that the activity of STAT3 mainly depends on the homodimerization of STAT3 and the activation of gene transcription, which regulates the proliferation, apoptosis, invasion and metastasis, angiogenesis and immune status in TME of colorectal cancer. IL-6 triggers the JAK-STAT3 pathway via IL-6R, which stimulates the production of miR935p in G-MDSC. This leads to a rise in the levels of miR935p in GM-EXO, resulting in enhanced infiltration of G-MDSCs and M2 macrophages. Empirical studies have demonstrated that the utilization of anti-IL-6R antibody in cases of prolonged inflammation and the application of STAT3 inhibitor (JSI-124) during dysplasia can effectively impede the development of CAC (Liu et al., 2018). IFN- λ 3 (IL-28B) exhibits both antineoplastic and immunomodulatory properties. In colon cancer, it can prevent the polarization of M2 macrophages by blocking the signal transduction factors and transcriptional activators STAT3 and JNK signal pathways (Gargalionis et al., 2021).

These small molecular preparations for cytokines are still in the experimental stage. We can try to improve the immune state, especially reversing the immunosuppressive state in TME, and resist drug resistance. Therefore the potential benefits of these medications for CAC patients are yet to be investigated.

#### 6.1.4 Inhibitors of PI3K/Akt/mTOR pathway

PI3K/AKT/mTOR pathway is a central mediator of inflammatory reponse and the sensor of the cell metabolism because the mTOR integrates growth factor signals with cell nutrition and energy levels to coordibates cell growth, proliferation and survival (Angevin et al., 2014; Covington et al., 2020). Activation of PI3K/AKT/mTOR will increase drug resistance of colorectal cancer cells, inhibits apoptosis, and promotes tumor cells survival. Besides, this pathway will produce higher levels of free fatty acids to create an immunosuppressive TME which suppresses immune checking, suggesting that the combination of PI3K-AKT-mTOR inhibition and checkpoint blocking may be a promising approach (Wang and Zhang, 2014; Wei et al., 2019). Therefore, inhibitors of this pathway may be beneficial to the treatment of CAC in terms of immune metabolism.

Given that the response to PI3K inhibitors is best when PIK3CA or PTEN is mutated, the incidence of which is less than 20%, PI3K inhibitors may be more effective in combination with other treatments (Palsson-McDermott and O’Neill, 2020). BKM120 is an irreversible PI3K inhibitor, which blocks PI3K by covalently modifying Lys-802 residues involved in phosphate transfer reaction, thus inhibiting the growth of cancer cells and has anticancer effect (O’Donnell et al., 2018). Rapamycin is a specific inhibitor of mTOR1, which could significantly impact the therapy of CAC. Rapamycin, a natural inhibitor of mTOR, has demonstrated the ability to hinder the proliferation of certain tumors and trigger apoptosis. Suppressing the activity of mTORC1 (Raptor) and mTORC2 (Rictor) hindered the movement and infiltration of CRC (Yang et al., 2021). Torin-1, a potent and specific inhibitor of mTOR, has been demonstrated to be the most efficacious inhibitor capable of suppressing the proliferation of metastatic cancer cells (Kim and Eng, 2012). AZD-2014, a potent inhibitor of both mTOR1 and mTORC2, effectively suppressed the activation of mTORC1 and mTORC2 and induced autophagy in colorectal cancer cells, resulting in the inhibition of their proliferation (Papadatos-Pastos et al., 2015). Aspirin-induced inhibition of mTOR depends on the independent-AMPK and dependent-AMPK mechanism. The concern about mTOR inhibition is the possibility of Akt activation triggered by feedback. The main cellular response induced by aspirin is mTOR inhibition rather than Akt activation. Aspirin and metformin (the activator of AMPK) increased the inhibition of mTOR and Akt, as well as autophagy of CRC cells (Gulhati et al., 2011).

#### 6.1.5 Agents targeted TLR4/NF-kB

TLR4 inhibitor TAK-242 was used to inhibit TLR4 signal transduction in colonic epithelial cells. Tumorigenesis of CAC can be prevented by using TAK-242 during intestinal inflammation via preventing macrophage infiltration. However, TLR4 receptor inhibitors may lead to increased susceptibility to infection and impaired wound healing and other side effects. By blocking the S100-PRR interaction, rCT-S100A8/9 can improve colitis while retaining the TLR4-dependent antibacterial defense mechanism, which may become a potential therapeutic target for CAC (Riddell et al., 1983; Francipane and Lagasse, 2013).

A selective class IIa histone deacetylase (HDAC) inhibitor TMP195 exerts its anti-colorectal cancer effect by promoting the polarization of M1 macrophages and enhancing the role of PD-1 blockers. Besides, TMP195 can decrease the activation of NF-kb and excessive inflammation by reducing the deacetylation of p65 at lysine-218 (Li et al., 2009; Huozhong et al., 2014). Phosphoinositide-3-kinase regulatory subunit 3 (PIK3R3), a member of the PI3K family, is upregulated in colonic tissues of both CAC and IBD patients. It inhibits the expression of intestinal tight junction protein, especially ZO-1, and increases intestinal permeability by activating NF-Kb. TAT-N15, a specific inhibitor of PIK3R3, can inhibit intestinal inflammation and is a new therapeutic target for IBD and CAC (Abdel-Aziz et al., 2014). NF-KB continued to increase in the development of CAC, but the expression of ITF2 increased in dysplastic tissues, but disappeared in cancer tissues, indicating that NF-kB was upregulated in dysplasia and was further activated after ubiquitin decomposition of IFT2 in carcinoma stage. Therefore, ITF2 may be a powerful target against canceration of colon tissue (Wu et al., 2018).

TLR4/NF-kB signalling pathway plays a crucial role in generating inflammation that contributes to the development of cancer and is strongly linked to the body’s immune response. Further research is needed to determine how to effectively manage it without compromising the body’s antibacterial immunity.

### 6.2 Drugs for cell death

#### 6.2.1 Drugs for autophagy

The precise contribution of autophagy to the progression of CAC is uncertain, and the optimal utilization of autophagy in CAC therapy remains undetermined.

A number of drugs have been used to inhibit autophagy, including 3-methyladenine (3-MA) (Aksan et al., 2021), chloroquine (CQ) (Hu et al., 2022), bafilomycin-A1 (Sun et al., 2021), AZD-2014 3-MA can enhance the 5-FU-induced apoptosis in colorectal cancer cells. CQ enhances the cytotoxicity of Sunitinib in a synergistic manner by inducing apoptosis and shutting down the mechanisms of autophagy and angiogenesis. Bafilmycin-A1 (BAF-A1) enhances the apoptosis induced by pyrrole-1, 5-benzoxazine (PBOXs) by reducing the level of anti-apoptotic protein Mcl-1. AZD-2014 promotes autophagy death by blocking the activation of mTOR, thus inhibiting the growth of tumor cells.

#### 6.2.2 Iron supplementation

Ferroptosis activation has the ability to not only inhibit colorectal cancer, but also to overcome conventional resistance to tumour drugs.

Appropriate iron supplementation is beneficial to the treatment and prognosis of colon tumor patients (Ogando et al., 2019). The GSH/GPX4 axis is considered to be the main system suppressing ferroptosis. Therefore, GCH/BH4 inhibitors are also a new treatment strategy, such as the combination of GCH1 inhibitors and eastin (Chang et al., 2015). The dichloroacetate (DCA) decreased the viability of CRC cells and showed anticancer effect by up-regulating iron levels (Ganesh, 2022).

#### 6.2.3 Immune checkpoint blockade inhibitors

Immune checkpoint inhibitors (ICI) can block the an-tumor immunity in TME and promote the immune cells to kill tumor cells (Lu et al., 2021). Some studies have demonstrated that blockade of immune checkpoint can inhibit the glycolysis of tumor cells, avoid T cells to use the restored glucose in TME and promote the production of cytokines (Keating, 2012).

Furthermore, ICI therapy is typically administered to patients who have advanced metastases. Pembrolizumab, an anti-PD1 monoclonal antibody, is utilized as an initial therapeutic option for CAC characterized by high microsatellite instability (MSI-H) and deficient mismatch repair. DMMR (Bai et al., 2021). For MSS colorectal cancer, it has been proved that combined blocking of IL-17A and PD-1 can better block the occurrence of colorectal cancer (An et al., 2017).

### 6.3 Herbal medicine

#### 6.3.1 Dihydroartemisinin

Dihydroartemisinin (DHA) is a derivative of artemisinin, and acts as an anti-malarial drug with both anti-inflammatory and anti-tumor effects (Simiao et al., 2020; Li et al., 2021; Qin et al., 2021). Oral DHA inhibits the development of CAC by inhibiting macrophage-related inflammatory response in the early stage, including reducing the expression of cytokines, down-regulating the phosphorylation of ERK, STAT3 and NF- kB p65, and inhibiting the growth of tumor cells in the late stage (Qin et al., 2021).

#### 6.3.2 Arctigenin and Atractylenolide

Arctigenin inhibited the expression of proinflammatory cytokines especially IL-1 β, in colonic macrophages of cancer patients by inhibiting the inflammatory activation of NLRP3. During the assembly of NLRP inflammatory bodies, arctigenin downregulate fattyacidoxidation (FAO), but not glycolysis, in macrophages (Yan et al., 2022).

Atractylenolide I is a highly effective active component in Atractylodes macrocephala, which has obvious inhibitory effects on many kinds of tumor cells. Atractylodes I inhibits the activation of NLRP3 inflammatory bodies in colitis-associated colorectal cancer by inhibiting Drp1-mediated mitochondrial division (Stine et al., 2022).

#### 6.3.3 Berberine

Researchers have found that berberine (BBR) plays an anti-colon cancer effect through a variety of signaling pathways. For instance, BBR can induce apoptosis, autophagy, and cell cycle arrest in colon tumor cells by blocking the PI3K/AKT/mTOR pathway (Phan et al., 2014). Furthermore, it has been discovered that BBR mitigates the development of CAC by increasing the abundance of bacteria that create short chain fatty acids, while simultaneously decreasing the presence of infections ^[158]^.

#### 6.3.4 Dimethyl itconate

Itconate derivative, dimethyl itconate (DI), can reduce the recruitment of macrophages and the production of MDSCs in TME by inhibiting the secretion of cytokine IL-1 β by intestinal epithelial cells which makes intestinal cells and mesenchymal cells secrete a large amount of chemokine CCL2, thus reducing the ratio of CD4/CD8, reducing the high inflammatory state of ulcerative colitis and reducing the risk of CAC ^[159]^.

### 6.4 Agents targeted metabolism

Glycolysis inhibition in tumor may be an effective treatment opportunity. For example, STF-31 is a small molecule inhibitor of glucose transporter 1, which can inhibit the uptake of glucose in tumor although it may have off-target effect. Glutor and Bay-876 can reduce the glycolytic flux by inhibiting glucose transporter protein. Besides, Inhibition of lactate dehydrogenase can reduce the conversion of lactate to pyruvate in tumor cells ^[160]^.

Amino acid metabolic reprogramming also occurs frequently in tumors. For example, glutaminolysis is enhanced in many tumors. Glutamine can be converted into α-KG to participate in the TCA cycle of tumor cells, so inhibition of glutamine intake through ASCT2 and c-myc, an inducer of glutamine decomposition, may be a promising anti-cancer metabolic method. ^[161]^ IDO can inhibit the proliferation of functional T cells and natural killer cells and promote the differentiation and activation of Tregs. Therefore, the use of IDO inhibitor, 1-methyltryptophan (1-MT), can restore anti-tumor immunity and improve CAC (Rokavec et al., 2014).

## 7 Conclusion

Individuals diagnosed with IBD face a growing likelihood of acquiring CAC, which is associated with a worse prognosis. When compared to sporadic CRC, CAC appears to be more inactive, leading to difficulties in timely detection and treatment. Furthermore, the challenges of agent usage are compounded by both drug resistance and the intricacy of immune control.

CAC, being an immune-mediated disease, can be linked to the emerging topic of “immunometabolism”. The immune system is an intricate network in which multiple signaling pathways and cytokines interact, playing both beneficial and harmful roles in the several phases of CAC. These stages involve metabolites, macrophage polarization, and the functional change of immune cells.

Anyway, additional study is needed to explore methods for preventing the onset and impeding the progression of CAC. Here, agents are focused on addressing anti-inflammation, immunological modulation, and the metabolism of immune cells and tumor cells.

## Figures and Tables

**Table:** 

**CRC**	colorectal cancer
**CAC**	colitis associated colorectal cancer
**IBD**	inflammatory bowel disease
**LGD**	low-grade dysplasia
**HGD**	high-grade dysplasia
**TME**	immunosuppressive tumor microenvironment
**TCA**	Tricarboxylic acid
**AD**	Alzheimer’s disease
**AMPK**	adenosine monophosphate–activated protein kinase
**AIDs**	Autoimmune diseases
**IRG1**	immune response gene
**SDH**	succinate dehydrogenase
**ROS**	reactive oxygen species
**PKM2**	pyruvate kinase M2
**iNOS**	inducible nitric oxide synthase
**VEGF**	vascular endothelial growth factor
**sLZIP**	leucine zipper protein
**NrF2**	nuclear factor erythroid2-related factor 2
**DAMPs**	damage-associated molecular patterns
**CTLs**	cytotoxic T lymphocytes
**mTOR**	mechanistic target of rapamycin
**TAM**	tumor associated macrophages
**a-KG**	a-ketoglutarate
**SLC7A2**	Solute carrier family 7 member 2
**L-Arg**	L-arginine
**MDSCs**	myeloid-derived suppressor cells
**CCL2**	C-C motif chemokine ligand 2
**G-MDSC**	granulocytic MDSCs
**M-MDSC**	Monocytic MDSCs
**IDO**	indoleamine 2,3 dioxygenase
**Kyn**	Kynurenine
**EMT**	Epithelial to mesenchymal transition
**Fra-1**	fos-related antigen-1
**TLR4**	Toll-like receptor
**SCFAs**	short-chain fatty acids
**ETBF**	enterotoxigenic *bacteroides fragilis*
**Fn**	*Fusobacterium* nucleatum
**5-ASA**	5-Aminosalicylates
**AT-SPMs**	aspirin-triggered specialized proresolving mediators
**PD-1**	programmed cell death protein-1
**MSS**	microsatellite stable
**HDAC**	histone deacetylase
**PIK3R3**	Phosphoinositide-3-kinase regulatory subunit 3
**3-MA**	3-methyladenine
**CQ**	chloroquine
**BAF-A1**	Bafilmycin-A1
**PBOXs**	pyrrole-1, 5-benzoxazine
**DCA**	dichloroacetate
**ICI**	immune checkpoint inhibitors
**MSI-H**	high microsatellite instability
**DHA**	Dihydroartemisinin
**FAO**	fattyacidoxidation
**BBR**	berberine
**DI**	dimethyl itconate
**1-MT**	1-methyltryptophan
